# Dynamic Price Application to Prevent Financial Losses to Hospitals Based on Machine Learning Algorithms

**DOI:** 10.3390/healthcare12131272

**Published:** 2024-06-26

**Authors:** Abdulkadir Atalan, Cem Çağrı Dönmez

**Affiliations:** 1 Department of Industrial Engineering, Çanakkale Onsekiz Mart University, Çanakkale 17100, Turkey; 2 Department of Industrial Engineering, Marmara University, Istanbul 34854, Turkey; cem.donmez@marmara.edu.tr

**Keywords:** show-up, no-show, appointment system, machine learning, dynamic price policy

## Abstract

Hospitals that are considered non-profit take into consideration not to make any losses other than seeking profit. A model that ensures that hospital price policies are variable due to hospital revenues depending on patients with appointments is presented in this study. A dynamic pricing approach is presented to prevent patients who have an appointment but do not show up to the hospital from causing financial loss to the hospital. The research leverages three distinct machine learning (ML) algorithms, namely Random Forest (RF), Gradient Boosting (GB), and AdaBoost (AB), to analyze the appointment status of 1073 patients across nine different departments in a hospital. A mathematical formula has been developed to apply the penalty fee to evaluate the reappointment situations of the same patients in the first 100 days and the gaps in the appointment system, considering the estimated patient appointment statuses. Average penalty cost rates were calculated based on the ML algorithms used to determine the penalty costs patients will face if they do not show up, such as 22.87% for RF, 19.47% for GB, and 14.28% for AB. As a result, this study provides essential criteria that can help hospital management better understand the potential financial impact of patients missing appointments and can be considered when choosing between these algorithms.

## 1. Introduction

The appointment system in the healthcare system plays a critical role for many vital reasons [1]. Generally, appointment systems belonging to sub-units of health institutions regulate and organize the functioning of hospitals and clinics [2]. It collects patient flow, plans staff duties, and ensures patients receive regular examinations or treatment. The appointment system helps the hospital use its resources more efficiently. Appointments enable staff and facility resources to be allocated more effectively. The appointment system prevents patients from waiting for long periods. This helps patients receive examination or treatment more quickly and access healthcare more efficiently. The appointment system distinguishes between emergencies and routine examinations [3]. Emergencies can be handled more quickly, while other patients are scheduled according to their appointment times. The appointment system helps patients have a more positive experience with healthcare [4]. Decreasing waiting times, providing appointment data at more convenient times, and the opportunity to receive a more regular service increase patient satisfaction.

The appointment system provides the opportunity to collect essential data to monitor and evaluate the use of health services [4,5]. The collected data can be used to assess hospital or clinic performance, plan, and direct resources. Hospital units with an appointment system can send reminder messages or calls to prevent patients from forgetting their appointments [6]. This reduces appointment losses and makes treatment processes uninterrupted. As a result of all these issues, the appointment system is a critical element in the healthcare system that increases efficiency, organization, patient access, and satisfaction. Hospital appointment systems play an essential role for healthcare providers and patients.

Many factors can affect patients’ show-up and no-show status in the appointment system [7]. Factors such as reminders, available appointment times, wait times, patient education, and procedure quality affect patients’ likelihood of attending their appointments [8,9]. At the same time, economic conditions, health insurance, and transportation also cause patients to miss their appointments [10,11]. Health systems develop effective strategies by taking these factors into account to increase patients’ “show-up” situations and reduce “no-show” situations [8]. The proper functioning of appointment systems affects health outcomes and patient satisfaction by facilitating patients’ access to regular health services [12].

The machine learning (ML) method is among today’s indispensable applications due to the concrete results obtained by applying it in many areas such as education, economy, transportation, health, and production [13,14]. In particular, the relationship between ML and healthcare systems is becoming increasingly important [15]. ML has excellent potential in several applications in the healthcare industry, such as data analysis, health insurance plans, disease diagnosis, development of treatment methods, monitoring of patients, and management of healthcare services [16,17]. By analyzing large amounts of health data, ML algorithms can help achieve important goals such as the early diagnosis of diseases, personalizing treatment plans, and improving patient outcomes [18]. Additionally, ML techniques are used to effectively manage patient care, use resources more efficiently, and improve the overall performance of healthcare systems [19]. Therefore, collaboration between ML and healthcare systems fosters innovations that benefit patients and healthcare providers.

Today, ML algorithms enable significant innovations in many fields, including healthcare. ML algorithms’ distributed federated learning perspective increases healthcare systems’ performance by providing faster and more efficient learning processes, especially on large data sets [20]. This approach allows for more comprehensive and reliable predictions to be obtained by collecting data from different sources and combining them in a central model. Moreover, the Levenberg–Marquardt neural network-based lifetime extension approach optimizes the lifetime of devices and systems [21]. The neural network algorithm improves the maintenance and performance monitoring of medical devices used in healthcare, reducing costs and increasing service quality [22]. These literature studies demonstrate how machine learning innovations can be applied in healthcare and the potential benefits of these applications.

The relationship between the patient appointment system and ML is essential for managing healthcare services more effectively and efficiently and improving patient’s access to healthcare [23]. ML is used to optimize scheduling and tracking patient appointments, direct appointment reminders, and increase the likelihood of patients showing up for their appointments [24]. At the same time, ML is used to predict patients’ propensity to miss appointments and develop strategies based on this information [25]. In this study, show-up and no-show situations of patients with appointments in a hospital were predicted by using ML algorithms. Such predictions can help healthcare institutions and hospitals use resources more effectively and provide better patient care. Therefore, patient appointment systems and ML combine to improve healthcare efficiency and meet patients’ needs [26].

The scientific aspect of this article and the innovations it will bring to science further emphasize the potential and importance of using ML algorithms in the healthcare sector. This study went beyond traditional appointment systems and integrated ML algorithms to predict patients’ appointment attendance. In particular, using algorithms such as RF, GB, and AB represents an important step in this context. This approach can increase the healthcare system’s efficiency by enabling providers to manage resources more effectively and optimize patient care.

This study applies a dynamic pricing policy for polyclinics operating with an appointment system using data from a non-profit hospital. Public hospitals, research and practice hospitals, and healthcare institutions operating within the scope of social responsibility projects are necessary healthcare service providers that are generally non-profit but aim to meet the general health needs of society [27]. Such hospitals provide preventive health services such as disease prevention, screening, and vaccinations, focusing on improving public health [28]. In addition, while research and practice hospitals aim to educate medical students and other health professionals and contribute to health research, hospitals within social responsibility projects generally strive to help their societies from a broader perspective [29]. When evaluating these hospitals, it is necessary to take into account critical elements such as financial sustainability, contribution to public health, support for education and research, social impacts, accessibility, and service quality to understand the crucial role of these organizations in the field of healthcare.

The dynamic pricing method proposed in this study offers significant advantages in preventing hospital financial losses. Predicting patients’ appointment attendance using ML algorithms allows hospital management to use their resources more efficiently. In particular, using algorithms such as RF, GB, and AB provides for a more accurate prediction of patient behavior and optimizing the appointment system accordingly. This approach will provide a scientific basis for determining the penalty pricing policy to be applied if patients do not attend their appointments. As a result, this innovative method can potentially improve patient satisfaction and the accessibility of healthcare services while increasing the financial sustainability of hospitals.

This article consists of five main sections. The first section introduces the article by introducing the importance of healthcare appointment systems and the relationship between ML and healthcare. The second section describes the data collection methods, the ML algorithms used, and the analysis procedure. The third section presents the performance results and analysis of the algorithms used. Information is given about the dynamic pricing policy developed to prevent losses due to patients not showing up during the appointment periods. The fourth section discusses this study’s general results and the implications of the proposed method for application, and it includes the study’s limits. The final section summarizes the study’s results and provides information about its contributions to future research.

## 2. Materials and Methods

### 2.1. Data Compilation

**Figure 1:**
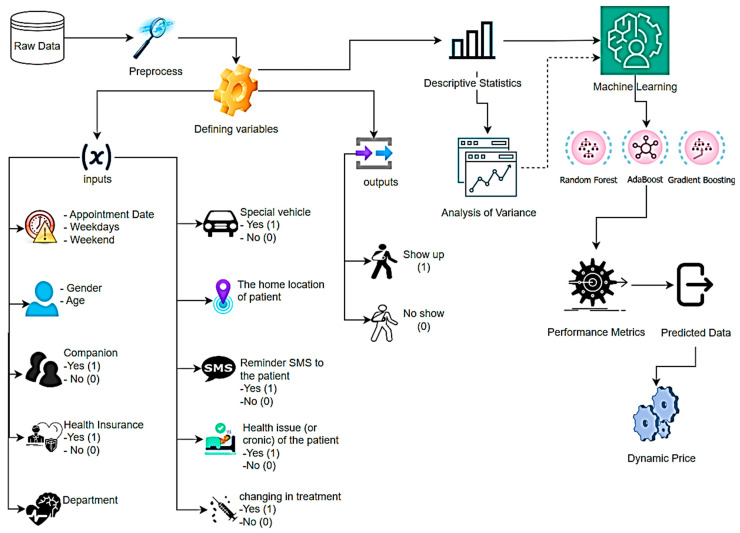
The flowchart of the methodology for the research.

This study aimed to improve healthcare and patient care management by running ML algorithms on the appointment status of 1073 patients in a hospital. Thirteen independent variables were used to identify factors affecting patients’ appointment behavior, optimize appointment reminders, and implement a dynamic pricing policy. Independent variables were defined as appointment date (seasonally), patient gender, working days of the week, weekends, patient age, health insurance, companion status, private vehicle ownership, health problem, patient’s residence, permanent disease status, and treatment change during the treatment period. This study also analyzed variance to examine the effects of the defined independent variables on the dependent variables, show-up and no-show situations. The main reason for determining these variables is that they have uninterrupted data sets and are variables that are frequently emphasized in the literature. The method flow of the study is shown in Figure 1.

Data from patients who made appointments for nine different departments were considered in this study. There are many reasons for using patient information from different polyclinics. First, the different health resources employed in these departments may affect the appointment system. Another reason is that the treatment times planned for patients in these departments are different, causing different appointment slot times in the appointment system. Finally, different departments must be considered to determine whether patients comply with their appointments depending on their disease type. Descriptive statistical data of patient data received from the hospital in terms of gender and departments are given in Table 1.

There are 1073 total patients, 706 of whom are female and 367 are male. While females constitute a large proportion of the total number of patients, males constitute a smaller group. The distribution of patients varies between treatment departments. The highest average number of patients is in the radiology department (101), and the lowest is in the hematology department (38). Statistically, the average number of patients in departments varies slightly depending on the gender of the patients. While more male patients (36) go to the orthopedics department, there are more female patients (66) in the hematology department. Standard deviation and variance values, which indicate how much variance the distribution of patients shows between departments, reflect the heterogeneity of these departments. The standard deviation in the radiology department is higher than the others, indicating that the data distribution in this department is more variable. The skewness value indicates whether a distribution is skewed to the right or left. Negative skewness values suggest that the distribution is skewed to the left, while positive skewness values indicate skewness to the right. Negative skewness values are seen in all sections, meaning the distributions are skewed to the left, not the right. As a result, while differences are observed between treatment departments, the distributions between genders also differ in certain departments.

Factors such as appointment date, time, weekday, or weekend have a decisive impact on patients’ attendance. These factors are important variables that affect patients’ attendance at their appointments. Depending on the appointment date, seasonal differences, weather conditions, and holiday periods may affect attendance. Weekday and weekend appointments may also lead to different results, as patients’ work schedules and daily life patterns may be associated with these factors. Therefore, appointment dates and times are essential for healthcare providers to consider when scheduling appointments. The patient’s age, gender, and the presence of a companion have significant effects on patients’ appointment attendance. A patient’s age is often a determining factor in access to healthcare and appointment attendance. Age may affect patients’ susceptibility to health problems and their likelihood of attending appointments. Gender may influence attitudes toward healthcare and appointment attendance; females often demand more healthcare. A companion’s presence can make it easier for patients to attend their appointments, especially for elderly or care-dependent patients. These factors are essential for healthcare providers to consider when evaluating patients’ appointment attendance.

Health insurance can significantly impact patients’ access to healthcare and their likelihood of attending appointments [30]. Insurance can help patients cover treatment costs and have easier access to healthcare. Patients with health insurance may only participate in appointments due to treatment costs. Therefore, having health insurance may have a positive impact on appointment attendance. In this study, health insurance status was considered as an input factor. The private vehicle factor was defined as input in this study to investigate the effects of patients’ transportation methods to the hospital on appointment attendance. Patients who own a private vehicle can increase their likelihood of attending their appointments because going to the hospital with their vehicle is an easier and more accessible option. These patients can have more control over getting to their appointments on time and are less likely to miss appointments due to unexpected transportation issues. On the other hand, patients who have to travel to the hospital by public transportation or on foot may need help reaching their appointments. Transportation issues can make patients more likely to miss appointments.

A reminder SMS is an essential factor that positively impacts patients’ show-up and no-show status in their appointments. These SMS reminders prevent patients from forgetting their appointments and encourage them to attend them on time. Therefore, sending SMS reminders to patients can be an effective strategy for healthcare providers to increase appointment attendance and streamline treatment processes. This factor is seen as an essential tool to encourage patients to attend their appointments regularly and improve the effectiveness of healthcare services.

Another critical factor is the distance of the patients’ home locations to the hospital. The distance of patients’ home locations to the hospital is an essential factor in terms of access to healthcare and may affect whether patients attend appointments. This factor determines the ease or difficulty of patients reaching healthcare facilities. Patients nearby may attend appointments more efficiently, while those who live in remote areas may have more difficulty accessing healthcare. Long distances and transportation problems can cause them to need help getting to appointments. This may lead to appointment cancellations or increased non-attendance rates. In connection with this factor, whether the patients had personal vehicles was also evaluated.

Another input factor considered in this study is whether patients’ chronic conditions significantly impact appointment attendance. Patients with chronic conditions may be more likely to attend their appointments due to their need for regular medical monitoring and treatment. These patients may be able to participate in appointments to monitor their health and adhere to their treatment plans. Additionally, patients may value these appointments since chronic conditions often require regular health checks.

Another factor is the sensitivity of patients whose treatment has changed during long treatment periods to appointments, which was investigated in this study. There are two full-fledged hospitals in the location where the study data are considered. The population in this location is approximately 87000. There are four types of transportation to the hospital: walking, public transit, private vehicles, and ambulance. Show-up and no-show situations are discussed in detail in this study, taking into account other factors of the patients.

### 2.2. Machine Learning Algorithms

Machine learning (ML) is a branch of artificial intelligence that allows computer systems to learn from data, infer patterns and relationships, and use this information for future decisions or predictions [14]. ML analyzes data and recognizes and learns patterns in this data. Generally, ML consists of the following phases: data collection, data preprocessing, model selection, model training, model evaluation, and model deployment [31]. During the data collection phase, relevant data are collected. Data preprocessing includes data cleaning, organization, and feature engineering. Model selection requires determining the most appropriate ML algorithm and model. Model training involves teaching data being appropriately applied to the algorithm. Model evaluation allows for testing and improving the performance of the model. Finally, a successful model is deployed for use in the real world. In this way, ML extracts information from data in many application areas.

**Figure 2:**
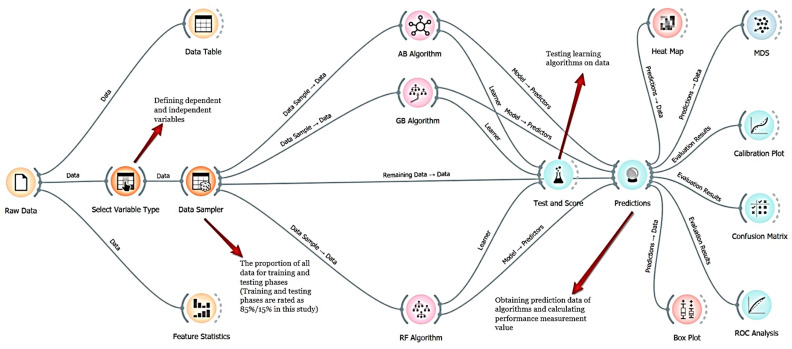
The flowchart of the ML algorithms for the present study.

In this study, patients’ show-up and no-show status was tried to be predicted using AB, GB, and RF algorithms, which are among the ML algorithms. The RF, AB, and GB algorithms were preferred because they are known for their high accuracy and balanced performance and they provide effective results, especially in complex data sets. These algorithms’ flexibility and powerful prediction capabilities also increase their applicability to prediction problems such as patient appointment prediction in healthcare. The workflow chart of ML algorithms is shown in Figure 2.

Adaboost (Adaptive Boosting—AB) is a machine-learning algorithm for classification and regression problems. Its basic idea is to combine weak learners (e.g., those with low accuracy) to create a strong learner [32]. The AB model adjusts the weights of data points, creates lean learners based on these weights, and trains them sequentially. Each new weak learner aims to obtain better results by focusing on the mistakes of previous learners. As a result, AB generally provides high accuracy and can be adapted to various problems thanks to its adjustable parameters. Therefore, it is a powerful and popular ensemble learning algorithm in classification and regression problems.

Gradient Boosting (GB) is a powerful ML technique for solving classification and regression problems. Its basic idea is to build a robust predictive model by sequentially combining weak learners (usually, decision trees are used) [33]. The main principle of GB is that each new tree is trained by focusing on the mistakes of previous trees. Each tree uses a specific percentage or feature of the data set and is best adapted to minimize the model’s errors. Therefore, GB generally provides high accuracy and can capture complex relationships in data. The GB model has different variations in multiple applications, such as XGBoost, LightGBM, and CatBoost, and can be adapted to many problems [34]. For this reason, Gradient Boosting is a popular option in many ML competitions and industrial applications.

Random Forest (RF) is an effective ML algorithm for classification and regression problems [35]. Its basic idea is to create a “forest” consisting of many decision trees and to obtain a more robust prediction model by combining the results of these trees. Each tree is trained with random data samples and random features, which increases the diversity of trees and reduces overfitting. RF consolidates the results by combining the predictions of each tree, thus achieving high accuracy. It can also be used to rank the importance of data features, making it possible to understand which features significantly impact prediction results [36]. Therefore, RF is an ensemble learning technique that can be used successfully in simple and complex problems and offers many applications.

Hyperparameter selection generally uses grid or random search methods in RF, GB, and AB models. In this process, the performance of different hyperparameter combinations was evaluated using cross-validation, and the most appropriate hyperparameters were selected to optimize a specific performance metric. Important hyperparameters vary depending on the characteristics of each algorithm, and regularization has also been considered to avoid overfitting. The selection of hyperparameters is often based on experimentation and experience, but it is also essential to understand the function and effects of each hyperparameter. The number of trees (n_estimators), the maximum number of features (max_features), tree depth (max_depth), and the minimum number of split samples (min_samples_split) for RF; the number of trees (n_estimators), learning rate (learning_rate), tree depth (max_depth), and the fraction of subsamples (subsample) for GB; and the number of trees (n_estimators) and the learning rate (learning_rate) for AB were taken into consideration to obtain the best performance on a given data set.

ML can encourage patients to attend appointments regularly and ensure their treatment processes are more effective and successful [37]. This technology offers a personalized approach using big data analytics to understand patients’ behaviors and trends better. Considering factors such as patients’ gender, age, chronic diseases, and past attendance behavior, they can direct their appointments via SMS reminders or personalized communication. Additionally, ML can help schedule appointments at more convenient times, thus increasing patient attendance. ML can monitor treatment processes and adapt them to patients’ needs. This may help develop more effective and efficient treatment methods and improve patients’ adherence to treatment. As a result, ML can be used as an essential tool to increase patient engagement and make treatment processes more successful.

Predicting whether patients will attend their appointments can help healthcare organizations manage resources more effectively and optimize treatment processes. There are advantages to using more than one ML algorithm when making this type of prediction. Various algorithms, such as AB, RF, and GB, offer different approaches and features. AB creates a robust prediction model by combining weak learners and copes well with imbalanced data sets. RF makes a powerful prediction model by combining various trees and provides the importance ranking of features. GB, on the other hand, offers high accuracy by merging trees sequentially. This diversity increases the likelihood that different algorithms will perform better for different data sets and problems. Additionally, by comparing the results of these algorithms, it is possible to choose the best prediction model and improve model performance. Therefore, combining different algorithms such as AB, RF, and GB effectively creates a more powerful and robust model for predicting patients’ appointment attendance.

### 2.3. Performance of ML Algorithms

This study calculated data for six performance measurement criteria: ROC (Receiver Operating Characteristic), CA (classification accuracy), F1, recall, precision, and MCC (Matthews Correlation Coefficient) [38]. AUC (area under the curve) refers to the area under the ROC. The ROC curve shows the relationship between sensitivity (recall) and 1-specificity (specificity). The AUC value ranges from 0 to 1 and measures the model’s classification performance. The closer it is to 1, the better the model’s performance. AUC measures the model’s accuracy rates and error rates. CA refers to the ratio of correctly classified samples to the total number of samples [39]. A high CA indicates the model’s ability to make accurate predictions [40]. However, it can be misleading in unbalanced data sets. CA expresses the ratio of correctly classified samples to the total number of samples and is calculated as follows:

(1) $$CA=\frac{\left(Correct\;Predictions\right)}{\left(Total\;Number\;of\;Samples\right)}$$

The F1 score refers to the harmonic mean of the precision and recall values of the model. It is helpful in unbalanced classification problems and deals with precision and incompleteness. The F1 score takes into account both false positives and false negatives. The F1 score is calculated as follows:

(2) $$F1=2\;*\frac{\left(Precision\;*\;Sensitivity\right)}{\left(Precision+Sensitivity\right)}$$

Precision is the ratio of the examples the model predicts as positive among the positive examples. It shows the ratio of true positives to total positive predictions. High precision indicates that there are few false positives. Precision is the ratio of samples that the model predicts as positive to those that are positive and is calculated as follows:

(3) $$Precision=\frac{\left(True\;Positive\right)}{\left(True\;Positive+False\;Positive\right)}$$

Sensitivity (recall) refers to the ratio of accurate positive samples among the total positive samples. It shows how many positive examples the model correctly predicted. High sensitivity indicates fewer false negatives. Sensitivity is the ratio of true positive samples to total positive samples and is calculated as follows:

(4) $$Sensitivity=\frac{\left(True\;Positive\right)}{\left(True\;Positive+False\;Negative\right)}$$

MCC (Matthews Correlation Coefficient): MCC is considered a balanced metric that measures the performance of the classification model. It evaluates the model’s success by considering true positive, true negative, false positive, and false negative values. As MCC approaches 1, it represents a perfect classification model. MCC is calculated using true positive (TP), true negative (TN), false positive (FP), and false negative (FN) values and is formulated as follows:

(5) $$MCC=\frac{\left(TP\;*\;TN-FP\;*\;FN\right)}{\sqrt{\left(TP+FP\right)*\;\left(TP+FN\right)*\;\left(TN+FP\right)*\;\left(TN+FN\right)}}$$

These formulas are used to measure the performance of the classification model, and each metric helps evaluate different aspects of the model. AUC measures the area under the ROC curve, while CA measures classification accuracy. The F1 score balances precision and sensitivity, while precision and sensitivity evaluate the accuracy of positive and negative predictions. As a balanced performance metric, MCC measures the success of the classification model from an overall perspective.

### 2.4. Dynamic Pricing Based on ML Algorithms

**Figure 3:**
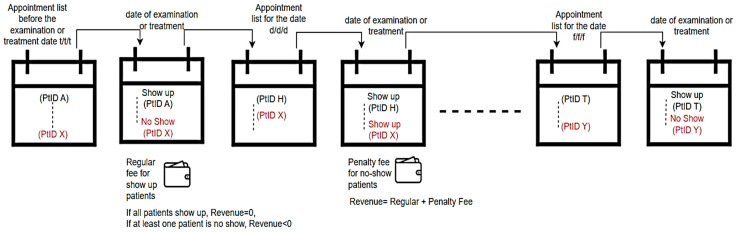
Workflow diagram of dynamic price application.

The price policies of the units of hospitals that work according to the appointment system are generally fixed. Still, the income status of the hospitals varies depending on whether the patients comply with their appointments. This study presents a dynamic pricing approach to prevent patients who have an appointment but do not show up to the hospital from causing financial loss. Dynamic price application is considered in this study only as an approach for patients who do not keep their appointments. The workflow for dynamic price implementation is shown in Figure 3.

Some assumptions for dynamic price application are expressed below:

The fee a patient must pay for treatment/examination (including fees such as co-payment): $P_{f}$

The number of patients appointed: $n_{t}$

The number of show-up patients: $n_{s}$

The number of no-show patients: $n_{n}$,

The following equation formulates the number of patients who do not show up:

(6) $${n_{n}=n}_{t}-n_{S}\;$$

The salaries of the healthcare personnel (physicians, nurses, technicians, etc.) employed in the unit where a patient applies for treatment/examination are paid by the hospital management on a salary basis. Personnel expenses are paid regardless of patient appointment. For this reason, this expense is defined as fixed. Resources cost (personnel): $c_{p}$.

Expenses for materials (equipment, medicine, consumables, etc.) the healthcare institution provides during a patient’s treatment/examination are variable costs. Material cost per patient: m.

$$material\;cost=\left\{\begin{array}{c} if\;patient\;is\;show\;up,\;\;\;\;\;\;\;\;\;\;\;\;\;\;\;\;\;\;\;\;\;\;\;\;\;\;c_{m}\\ if\;patient\;is\;no\;show,\;\;\;\;\;\;\;\;\;\;\;\;\;\;\;\;\;\;\;\;\;\;\;\;0 \end{array}\right.$$

We calculate the number of patients who did not come to their appointment but made a new appointment for treatment and examination (since these types of patients generally negatively affect the hospital’s income, it is discussed in this study that a penalty rate should be applied to them). The number of no-shows for the appointment but show up for the next appointment (reappointment): $n_{nr}$.

In this study, we calculated the number of patients who did not come to their appointments and, therefore, came for the first time due to the gaps in the appointment period and the fee received from the hospital management for this type of patient was taken into account as the first fee. This type of patient is included in the calculations so that no penalty or extra fee is charged. Number of new patients who made an appointment during the free appointment time slot: $n_{sn}$.

The limit of the number of new patients who make an appointment in the empty appointment slots and the number of patients who make a repeat appointment is determined by the formula below:

(7) $$n_{nr}+n_{sn}\leq \;n_{n}$$

The expected revenue $E_{r}$ of a hospital, taking into account patients subject to an appointment system, is given in the formula below:

(8) $${E_{r}=n}_{t}*{(P}_{f}-c_{m})-c_{p}$$

However, taking into account some patients who did not keep their appointments, new patients, and no-show patients who made new appointments to replace these patients, the expected revenue was reviewed, and the following equation was created:

(9) $${R_{r}=[(n}_{s}{+n}_{sn})*{(P}_{f}-c_{m})]+[n_{nr}*(\varphi -c_{m})]-c_{p}$$

where the penalty cost for $n_{nr}$ patients is defined as φ. Some limits for revised income need to be taken into account. The attendance rates of patients who did not attend their previous appointment and made a new appointment were evaluated using the confusion matrix values in the ML algorithms. Likewise, the hospital admission rates of new patients who found a place in the empty appointment slot were taken into account with the confusion matrix values. In the system proposed in this study, if patients who apply to the hospital for the first time do not come to their appointments, and if the same patients make an appointment for the second time, these patients will be subject to a penalty at the rate determined on the day of the appointment. However, if the same patients do not keep their appointments more than once, health services will be provided by increasing the penalty coefficients. The confusion matrix in Table 2 provides the patient numbers of these two patient types.

The probability of a patient who has not come to an appointment before making another appointment is considered as ${(1-\beta }_{i})$. The show-up status of a patient who makes a repeat appointment is calculated with the following formula:

(10) $$n_{nr}=\left(n_{t}-n_{ns}\right)*\left(1-\beta_{i}\right)*\beta_{i}$$

The number of times a new patient can make an appointment for an empty appointment slot is the following:

(11) $$n_{sn}=\left(n_{t}-n_{ns}\right)*\beta_{i}*\beta_{i}$$

Depending on the confusion matrix, the revised income was revised again, and the following equation was created:

(12) $${R_{r}=[n}_{s}*{(P}_{f}-c_{m})]+[\beta_{i}^{2}*(n_{t}-n_{ns})*{(P}_{f}-c_{m})]+[(\beta_{i}-\beta_{i}^{2})*(n_{t}-n_{ns})*(\varphi_{i}-c_{m})]-c_{p},\;i=\{RF,\;GB,\;AB\}$$

For the non-loss status of a non-profit hospital:

(13) $${[n}_{s}*{(P}_{f}-c_{m})]+[\beta_{i}^{2}*(n_{t}-n_{ns})*{(P}_{f}-c_{m})]+[(\beta_{i}-\beta_{i}^{2})*(n_{t}-n_{ns})*(\varphi_{i}-c_{m})]-c_{p}=0$$

The penalty cost value is the following:

(14) $$\varphi_{i}=\frac{P_{f}\beta_{i}^{2}n_{t}-P_{f}\beta_{i}^{2}n_{s}-\beta_{i}c_{m}n_{t}+\beta_{i}c_{m}n_{ns}+n_{ns}P_{f}-c_{m}n_{ns}-c_{p}}{\beta_{i}(\beta_{i}n_{i}-\beta_{i}\beta_{ns}-n_{s}+n_{ns}}$$

The penalty cost was applied only to patients who did not keep their appointments but rescheduled in the first 100 days. The outpatients were taken into account to observe the fluctuations in the penalty cost. 

## 3. Results

The results of this study were obtained from three different analyses and calculations. First, a statistical analysis of the factors affecting whether patients adhere to their appointments was performed. In the second stage, using the data of 1073 patients, it was estimated whether they would come to their next appointment or not as a result of analyzing whether the patients came to their appointments. To prevent financial losses caused by patients who do not comply with the hospital’s appointments, the mathematical approach developed for dynamic price application has enabled the formation of treatment and examination fees. This study used variance analysis to determine statistical significance between different groups or categories and to examine variance differences between groups. Analysis of variance data for the study variables is given in Table 3.

Important information showing that various factors affecting whether patients with appointments come to the hospital are statistically significant is shown in Table 3. The regression model represents the overall results of the variance analysis. In this analysis, we examine the relationship between the dependent variables show-up and no-show and the independent variables. There is a significant relationship between the date variable and show-up/no-show. If the *p*-value is less than or equal to 0.05, the independent variable considered is statistically significant on the dependent variable. The f-value is 9.9500, and the *p*-value is 0.002. This indicates that the date factor influencing whether patients attend their appointments is statistically significant.

There is also a significant relationship between the variable representing weekdays and weekends and show-up/no-show. The f-value is 38.770, and the *p*-value is 0.000. Whether it is a weekend or not significantly impacts appointment attendance. There is a strong relationship between age and show-up/no-show. The f-value is 131.87, and the *p*-value is 0.000. Age has a significant impact on whether patients attend their appointments. The statistical *p*-value of 0.05 for the age factor indicates that it is substantial for the dependent variable.

There is a significant relationship between vehicle ownership and show-up/no-show. The f-value is 1.0700, and the *p*-value is 0.023. This shows that patients’ ability to have a vehicle affects their likelihood of attending appointments. The relationship between companion and show-up/no-show is not statistically significant. The f-value is 2.1500, and the *p*-value is 0.097. This does not indicate that this variable significantly impacts appointment attendance. The relationship between insurance status and show-up/no-show is not statistically significant. The f-value is 0.0800, and the *p*-value is 0.190. It was analyzed that the effect of health insurance on the patient’s appointment was not statistically significant. There is a strong relationship between SMS reception and show-up/no-show. The f-value is 3431.7, and the *p*-value is 0.000. The likelihood of patients attending their appointment can vary significantly depending on whether they receive an SMS. There is a significant relationship between health problems and show-up/no-show. The f-value is 12.080, and the *p*-value is 0.001. If patients have health problems, it may affect their likelihood of attending their appointments.

The relationship between treatment change and show-up/no-show is not statistically significant. The f-value is 3.3200, and the *p*-value is 0.069. Changing treatment does not have a significant impact on appointment attendance. The relationship between episode selection and show-up is not statistically significant. The f-value is 0.5500, and the *p*-value is 0.058. There is no statistical significance regarding the part of the treatment received by the patient when attending the appointment. The relationship between citizenship status and show-up is not statistically significant. The f-value is 0.5000, and the *p*-value is 0.481. Citizenship status has little impact on appointment attendance. The relationship between location identity and show-up is statistically significant. The f-value is 2.4500, and the *p*-value is 0.000. Where patients receive treatment may affect appointment attendance. The relationship between gender and show-up/no-show is not statistically significant. The f-value is 0.0000, and the *p*-value is 0.962. Gender does not appear to have a significant impact on appointment attendance.

As a result, the analysis of various independent variables affecting patients’ show-up and no-show situations with appointments is discussed. Significant factors include Date, Wk/nd, Age, Own Vehicle Ownership, Receipt of SMS, and Health Problem. These factors can be considered statistically affecting patients’ attendance at their appointments. On the other hand, the factors of Insurance, Companion, Treatment Change, Department Selection, Citizenship Status, and Gender seem unimportant as they do not significantly impact show-up or no-show status.

Six performance measurement values (AUC, CA, F1, precision, recall, and MCC) were calculated to verify the validity of the results obtained from the ML algorithms applied to predict the appointment status of patients. Performance metrics play a critical role in evaluating and comparing how effective a model is. These measurements quantitatively assess the model’s predictive capabilities, accuracy, and overall performance and determine how successfully the model can solve real-world problems. Performance measurement values contribute to the effectiveness and reliability of algorithms by providing critical guidance in processes such as model selection, hyperparameter tuning, and general algorithm improvements. Table 4 contains the performance measurements of the training and testing phases of three different algorithms for show/no-show predictions of patients.

For the training phase, the performance measurement values of the algorithms were evaluated. The RF algorithm achieves a high AUC value (0.987) in the training phase. This reflects the model’s success in predicting patients’ appointments. It also shows high performance in CA, F1 score, precision, and sensitivity metrics. The MCC provides a very high balance of 0.975. RF shows that it creates a robust model during the training phase.

In the training phase, the GB algorithm achieves a high AUC value (0.984). CA also scores high in other metrics, such as F1 score, precision, and sensitivity. The MCC value provides a very high balance with 0.985. GB shows that it creates a robust model during the training phase. The AB algorithm achieves a high AUC value (0.989) in the training phase. It also exhibits high performance in other metrics, but its MCC value is slightly lower (0.947) than the other two algorithms. AB is developing a model that provides high accuracy during the training phase. In the testing phase, the RF algorithm still achieves a reasonably high AUC value (0.977). However, the CA, F1 score, and MCC value are slightly lower than the training phase. Still, the model performs well in predicting patients’ appointments. The GB algorithm also achieves a high AUC value (0.974) in the testing phase. It shows a balanced performance with an MCC value of 0.934. GB continues to make firm predictions during the testing phase.

In the testing phase, the AB algorithm achieves a slightly lower AUC value (0.939). CA, F1 score, and MCC value are slightly lower than the other two algorithms. However, the model still performs well in predicting patients’ appointments. As a result, the RF, GB, and AB algorithms generally perform well during the training and testing phases, although some metrics may drop slightly during the testing phase. All three algorithms develop powerful models to predict patients’ appointments. Table 5 shows the performance measurement values of the algorithms for show/no-show predictions of patients.

The RF algorithm has performed exceptionally well in predicting whether patients will attend their appointments. While the high AUC value (0.977) indicates that the model’s predictions are generally accurate, high scores were also obtained in essential metrics such as CA, F1 score, precision, and sensitivity—the MCC value also perfectly balances, indicating that the RF offers reliable and stable performance. The GB algorithm is also very effective in predicting patients’ appointments. A high AUC (0.974) shows that the model is booming, and other performance metrics such as accuracy, F1 score, precision, and sensitivity also have high values. The MCC value confirms that GB has a balanced performance.

The AB algorithm achieves a slightly lower AUC value (0.939) than the other two. However, it still provides good accuracy and sensitivity. Since the MCC value is slightly lower, AB’s performance is slightly more unbalanced than the others. As a result, the RF and GB algorithms perform very well in predicting patients’ appointments. Although AB has a slightly lower AUC and MCC than the others, it still offers good accuracy and sensitivity but has a more unstable performance.

**Figure 4:**
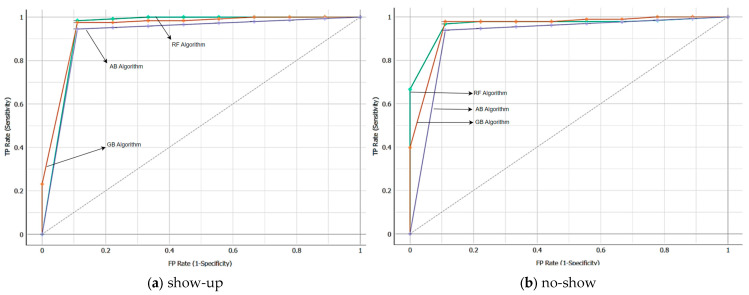
The ROC analysis of algorithms.

ROC analysis is an important tool used in ML and statistics to evaluate the performance of classification models and set decision boundaries. ROC analysis is used to assess the performance of a classification model. It summarizes the model’s accuracy, precision, specificity, and performance metrics. Additionally, ROC indicates the balance between a model’s sensitivity (true positive rate) and specificity (true negative rate). Thus, it helps us understand how the model achieves the balance between false positive and false negative predictions. The ROC analysis of the RF, AB, and GB algorithms used in this study is compared in Figure 4. In terms of ROC curves and AUC values, the RF model (0.987) shows better performance than the GB (0.974) and AB (0.939) models. AUC values of the algorithms were calculated according to the average values of the dependent variable type (no-show and show-up).

**Figure 5:**
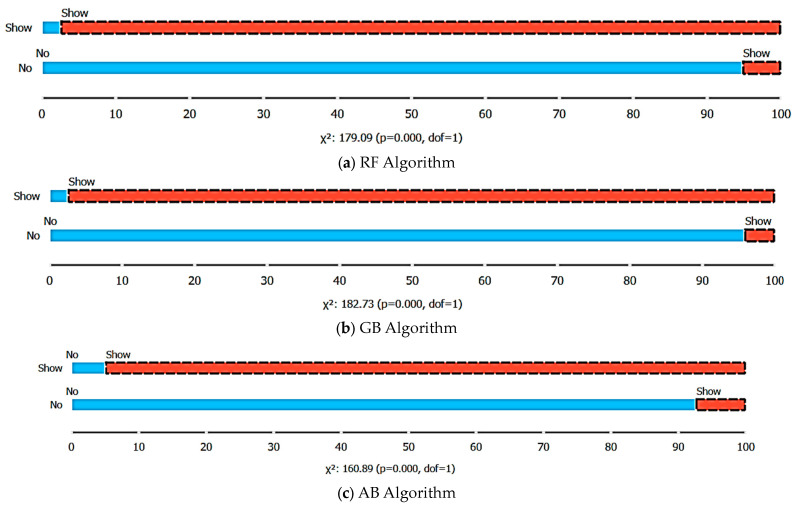
Box plots of algorithms (X^2^, chi-square; dof, degree of freedom).

Figure 5 presents box plots of prediction data for dependent variable types. The RF algorithm has an accuracy of 97.48% for the show variable and 94.74% for the no-show. The GB algorithm has an accuracy of 97.50% for the show variable and 95.74 for the no-show. Depending on the AB algorithm, the probability of patients being seen in the hospital is calculated as 95%. However, 92.55% accuracy was achieved in the prediction data of patients who did not keep their appointments (no-shows).

Table 6 shows the confusion matrix values containing the show or no-show predictions of patients made by three different classification algorithms (RF, GB, and AB). The confusion matrix describes the relationship between actual and predicted classes. These confusion matrices show how accurately each algorithm predicts true no-show and show-up patients. Although all three algorithms have high accuracy rates, there appear to be some inaccurate predictions. Confusion matrices are used to evaluate the performance of algorithms further and help evaluate performance metrics such as accuracy, sensitivity, and specificity. Confusion matrix data for both classes (no-show and show-up) are explained in detail for each algorithm.

RF correctly predicted 94.7% of the true no-show data, but 2.50% was incorrectly predicted as show-up. A total of 5.30% of the actual show-up data was incorrectly predicted by RF. A total of 97.5% of the actual show-up data was correctly predicted by RF. A total of 95.7% of the actual no-show data was correctly predicted by GB. A total of 2.50% of the actual no-show data was incorrectly predicted as show-up. A total of 4.30% of actual show-up data was correctly predicted by GB. A total of 97.5% of the actual show-up data was correctly predicted by GB. A total of 92.6% of the actual no-show data was correctly predicted by AB. A total of 5.0% of the actual no-show data was incorrectly predicted as show-up. AB correctly predicted 7.40% of the actual show-up data. AB has correctly predicted 95.0% of actual show-up data.

In ML, a calibration plot is a tool used to evaluate how well the model’s predicted probabilities match the probabilities of actual events. This graph helps us understand how reliable a model’s predictions are, especially in classification problems. A calibration plot is an essential tool in models working on probability predictions because it allows you to evaluate how accurately models predict classes and how reliable these predictions are.

**Figure 6:**
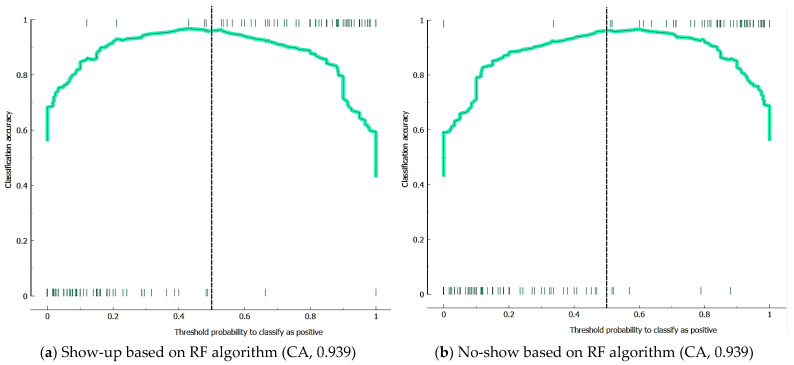

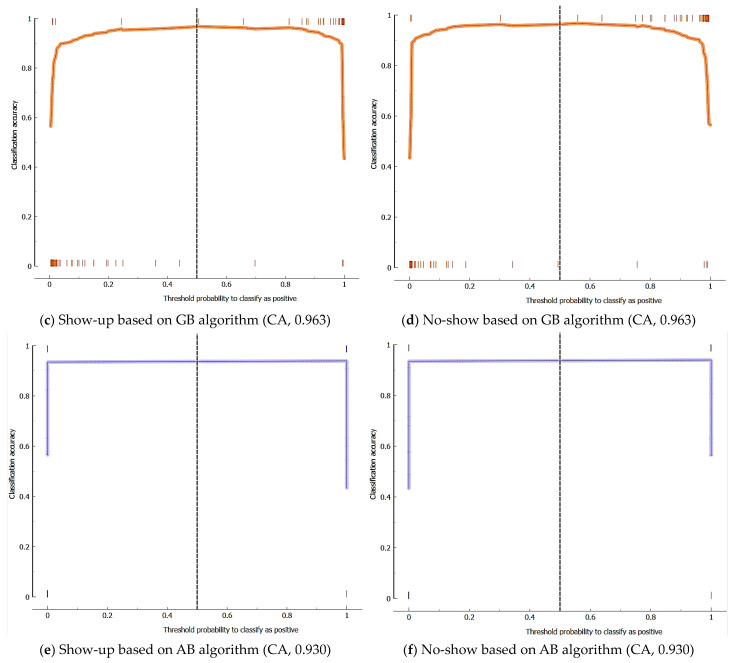
Calibration plots of algorithms.

The calibration plot’s *x*-axis (horizontal axis) is the average of the predicted probabilities or the predicted classification threshold values. The *y*-axis (on the vertical axis) shows the average of the probabilities of actual events or the percentage of real classes that were predicted correctly. A good calibration plot will be a curve representing a graph where the points are evenly distributed along that curve. This indicates that the model’s predictions are close to the true probabilities and that the model’s predicted probabilities are accurate. Calibration plots of the ML algorithms used in this study are shown in Figure 6.

In this study, the calculation of the income status of patients belonging to the units operating according to the appointment system of a hospital is discussed, taking into account their appointment follow-ups. Especially hospitals that are considered non-profit take into consideration not to make any losses other than seeking profit. For this reason, a model that ensures that hospital price policies are variable due to hospital revenues depending on patients with appointments is presented in this study. This study created a dynamic pricing policy using ML algorithms to predict patients’ show-up and no-show situations with appointments. By applying dynamic pricing, the hospital ensured that it did not incur losses.

**Figure 7:**
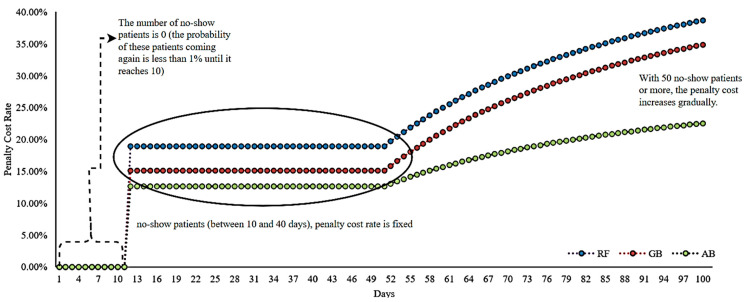
Penalty cost rates based on the ML algorithm.

For the revised revenue for the hospital, data from 1073 patients in an appointment system covering nine clinics were used. A 100-day data set was created by accepting the value representing the no-show rate of each ML algorithm as the maximum level. Depending on each ML algorithm, treatment/examination cost units are considered variable but fixed within themselves. One of the critical factors affecting the price adjustment in units connected to the appointment system is refilling the empty period caused by no-shows. New patients or no-show patients who make repeat appointments can fill free time slots. As the free period of the appointment system decreases, the penalty cost also decreases. According to this data set, the penalty cost rate increases as the number of vacant appointments increases. Penalty cost rates were calculated based on the performance measurement values of ML algorithms, with a calculation covering 100 days. Penalty cost rates for ML algorithms are shown in Figure 7.

Average penalty cost rates calculated based on three different ML algorithms used to determine the penalty costs that patients will face if they do not come to their appointments, such as 22.87% for RF, 19.47% for GB, and 14.28% for AB, reflect the diversity in the performance of the algorithms. This diversity provides important criteria that can help hospital management better understand the potential financial impact of patients missing appointments and can be considered when choosing between these algorithms.

## 4. Discussion

This study comprehensively evaluated the predictive performance of the RF, GB, and AB algorithms in forecasting patient appointment attendance in a hospital setting. The RF model exhibited superior performance with an AUC of 0.987, demonstrating its robustness in distinguishing between show-up and no-show instances. The GB algorithm also performed admirably with an AUC of 0.974, while the AB model, though slightly less effective, still provided reliable predictions with an AUC of 0.939.

Our analysis identified key factors influencing patient attendance, including appointment date, day of the week, age, and SMS reminders. The appointment date and day of the week had significant impacts, with patients showing a higher likelihood of attending weekday appointments. Age also played a crucial role, with younger and older patients attending more reliably than middle-aged individuals. The provision of SMS reminders emerged as a critical factor, significantly enhancing appointment adherence.

The dynamic pricing policy proposed in this study effectively mitigates financial losses due to missed appointments. By integrating the predictive capabilities of ML algorithms, the dynamic pricing model adjusts fees based on predicted attendance probabilities. This approach improves financial stability for non-profit hospitals and encourages patients to adhere to their scheduled appointments, optimizing resource utilization and reducing idle time.

These findings underscore the value of incorporating advanced ML techniques in healthcare management. The high predictive accuracy of RF and GB models, in particular, provides a reliable basis for implementing dynamic pricing and other strategic interventions to improve appointment adherence. Future research should explore the integration of additional variables and larger data sets to enhance predictive models further and develop more comprehensive strategies for managing patient appointments in various healthcare settings.

### Limitations

This article has some limitations. First, this study is based on a data set of patients only in a specific location. This means that the results may be limited in terms of generalization. Different results may be obtained in various geographical regions or different healthcare institutions. Therefore, the results of this study may need to be carefully evaluated before being applied to other healthcare providers or regions.

Second, although this study included some characteristics to predict patients’ appointment attendance, it has yet to address other potential factors. For example, patients’ personal socioeconomic status and education level may also affect appointment attendance. These factors are necessary for the model’s ability to provide a complete picture. Therefore, future studies may include more factors or use a more extensive data set to obtain more comprehensive and precise results.

A third limitation is that the data set of this study only covers a certain time. This means ignoring seasonal or temporal changes. Factors affecting appointment attendance may vary across different seasons or years. Therefore, using data covering a more extended period can help better understand seasonal variations and over-time trends.

The fourth limitation is that this study used only three different ML algorithms. Other potential ML approaches have yet to be noticed. Various algorithms may perform better for different data sets or problem types. Therefore, further comparative analyses on more ML algorithms may contribute to the development of better prediction models.

In addition, the hospital considered for this research, in which dynamic price application is recommended, gives effective results because it is the only hospital in the region. The dynamic pricing policy for areas with more than one hospital may need to be revised.

A final limitation is that the hospital is considered a public hospital type. Such hospitals incur financial losses due to patients not keeping their appointments. In addition to ensuring that the hospital taken into consideration in this study does not suffer financial losses, it is also taken into account that it is not for profit. For this reason, different assumptions should be considered when implementing a dynamic pricing policy for private healthcare institutions. Despite these limitations, this study represents an essential step toward improving the efficiency of healthcare services using the combination of hospital appointment systems and ML techniques.

## 5. Conclusions

This article creates a dynamic pricing policy for treatment or examination fees to prevent financial losses caused by patients who do not keep their appointments in non-profit health institutions. This study used three different methods to obtain the results in three steps. Analysis of variance was performed to measure the effects of independent factors affecting patients’ appointment status. In the second step, Adaboost (AB), Gradient Boosting (GB), and Random Forest (RF) models from machine learning (ML) algorithms were used to predict the appointment status of the patients. Performance measurement values were obtained to verify the validity of the prediction results of these algorithms. In the last step, the results of the dynamic pricing model were obtained, and a pricing calculation was made according to the occupancy and vacancy rates of the appointment schedule for the patients who did not keep their appointments in the next appointment period. It is understood that the dynamic pricing policy provides many advantages, such as helping healthcare organizations manage their appointment systems more effectively, providing better resource management, less idle time, and more fluid hospital operations.

Future contributions of this article include the potential for broader generalization through the use of larger data sets and data from different healthcare institutions. Additionally, by considering more factors, it will be possible for ML algorithms to develop more complex and precise models for predicting appointment attendance. This study may provide an essential basis for more effective management of healthcare services and better service to patients in the future. Using data from more healthcare institutions and larger data sets may increase the potential for broader generalization. In addition, many criteria, such as early appointment time, discounted treatment/examination fee, and extra service, should be discussed for patients who keep their appointments in future studies.

## Figures and Tables

**Table 1:** Descriptive statistical data of patient data based on gender and departments.

Variables	Departments	N	Mean	SEM	StDev	Variance	CoefVar	Skew	Kurt
Female	Allergy and Immunology	68	0.544	0.061	0.502	0.252	92.210	−0.180	−2.030
	Cardiology	80	0.488	0.056	0.503	0.253	103.180	0.050	−2.050
	Dermatology	85	0.518	0.055	0.503	0.253	97.100	−0.070	−2.040
	Gastroenterology	72	0.556	0.059	0.500	0.250	90.070	−0.230	−2.000
	Hematology	66	0.546	0.062	0.502	0.252	91.990	−0.190	−2.030
	Nephrology	76	0.461	0.058	0.502	0.252	108.950	0.160	−2.030
	Orthopedics	82	0.500	0.056	0.503	0.253	100.620	0.000	−2.050
	Radiology	101	0.446	0.050	0.500	0.250	112.110	0.220	−1.990
	Urology	76	0.474	0.058	0.503	0.253	106.110	0.110	−2.040
Male	Allergy and Immunology	43	0.488	0.077	0.506	0.256	103.560	0.050	−2.100
	Cardiology	44	0.477	0.076	0.505	0.255	105.860	0.090	−2.090
	Dermatology	41	0.585	0.078	0.499	0.249	85.210	−0.360	−1.970
	Gastroenterology	41	0.512	0.079	0.506	0.256	98.800	−0.050	−2.100
	Hematology	38	0.421	0.081	0.500	0.250	118.830	0.330	−2.000
	Nephrology	47	0.511	0.074	0.505	0.255	98.950	−0.040	−2.090
	Orthopedics	36	0.694	0.078	0.467	0.218	67.270	−0.880	−1.300
	Radiology	36	0.583	0.083	0.500	0.250	85.710	−0.350	−1.990
	Urology	41	0.463	0.079	0.505	0.255	108.940	0.150	−2.080
Total Count		1073 (Female, 706; Male, 367)							

Abbreviation: N, the total number of samples; SEM, standard error of the mean; StDev, standard deviation; Var, variance; CoefVar, coefficient of variance; Skew, skewness; Kurt, kurtosis.

**Table 2:** The confusion matrix for $n_{nr}$ and $n_{sn}$.

Algorithms	Proportion of Predicted % (Number of Patients)		
		Predicted No-Show	Predicted Show-Up
i	Actual No-Show	$\alpha_{i}$	$1-\beta_{i}$
	Actual Show-up	$1-\alpha_{i}$	$\beta_{i}$

**Table 3:** The analysis of variance of independent and dependent variables.

Source	DF	Adj SS	Adj MS	f-Value	*p*-Value
Regression	78	217.73	2.7910	55.000	0.000
Date	1	0.5050	0.5050	9.9500	0.002
Wk/nd (num)	1	1.9680	1.9680	38.770	0.000
Age	1	6.6930	6.6930	131.87	0.000
Own Vehicle	1	0.0540	0.0540	1.0700	0.023
Companion	1	0.1090	0.1090	2.1500	0.097
Insurance	1	0.0040	0.0040	0.0800	0.190
SMS received	1	174.18	174.18	3431.7	0.000
Health Issue	1	0.6130	0.6130	12.080	0.001
Treatment Change	1	0.1680	0.1680	3.3200	0.069
Departments	1	0.0280	0.0280	0.5500	0.058
Citizen or Not	1	0.0250	0.0250	0.5000	0.481
Location ID	66	8.2210	0.1250	2.4500	0.000
Gender	1	0.0000	0.0000	0.0000	0.962

Abbreviation: DF, degree of freedom; Adj, adjusted; Adj SS, adjusted sum of squares; Adj MS, adjusted mean of squares.

**Table 4:** The performance measurements of the training and testing phases of algorithms.

Stages	Algorithms	AUC	CA	F1	Precision	Recall	MCC
Train	RF	0.987	0.943	0.943	0.943	0.943	0.975
	GB	0.984	0.977	0.977	0.977	0.977	0.985
	AB	0.989	0.969	0.969	0.969	0.969	0.947
Test	RF	0.977	0.930	0.930	0.934	0.930	0.862
	GB	0.974	0.967	0.967	0.967	0.967	0.934
	AB	0.939	0.939	0.939	0.939	0.939	0.877

**Table 5:** The performance measurement values of the algorithms for show/no-show predictions.

Algorithms	AUC	CA	F1	Precision	Recall	MCC
RF	0.977	0.963	0.963	0.963	0.963	0.924
GB	0.974	0.967	0.967	0.967	0.967	0.934
AB	0.939	0.939	0.939	0.939	0.939	0.877

**Table 6:** The confusion matrix of algorithms.

Algorithms	Proportion of Predicted % (Number of Instances)		
		Predicted No-Show	Predicted Show-Up
RF	Actual No-Show	94.7% (90)	2.50% (3)
	Actual Show-up	5.30% (5)	97.5% (116)
GB	Actual No-Show	95.7% (90)	2.50% (3)
	Actual Show-up	4.30% (4)	97.5% (117)
AB	Actual No-Show	92.6% (87)	5.0% (6)
	Actual Show-up	7.40% (7)	95.0% (114)

## Data Availability

The data presented in this study are available on request from the corresponding author.
